# Inferential Costs of Trait Centrality in Impression Formation: Organization in Memory and Misremembering

**DOI:** 10.3389/fpsyg.2017.01408

**Published:** 2017-08-22

**Authors:** Ludmila D. Nunes, Leonel Garcia-Marques, Mário B. Ferreira, Tânia Ramos

**Affiliations:** ^1^Center for Instructional Excellence, Purdue University Lafayette, IN, United States; ^2^Centro de Investigação em Ciência Psicológica (CICPSI), Faculdade de Psicologia, Universidade de Lisboa Lisboa, Portugal

**Keywords:** impression formation, memory, DRM, centrality effect, organization

## Abstract

An extension of the DRM paradigm was used to study the impact of central traits (Asch, 1946) in impression formation. Traits corresponding to the four clusters of the implicit theory of personality—intellectual, positive and negative; and social, positive and negative (Rosenberg et al., 1968)—were used to develop lists containing several traits of one cluster and one central trait prototypical of the opposite cluster. Participants engaging in impression formation relative to participants engaging in memorization not only produced higher levels of false memories corresponding to the same cluster of the list traits but, under response time pressure at retrieval, also produced more false memories of the cluster corresponding to the central trait. We argue that the importance of central traits stems from their ability to activate their corresponding semantic space within a specialized associative memory structure underlying the implicit theory of personality.

## Introduction

As we use our memories and knowledge to navigate the world, our impressions of others' personalities are particularly important. The knowledge structure underlying impressions of personality has been dubbed *implicit theory of personality* (Bruner and Tagiuri, 1954). Further research was able to identify the organizing dimensions and the information content of this structure, and even account for fundamental impression formation effects (Rosenberg et al., 1968). However, the cognitive nature of this semantic structure was never made quite clear. Recently, Garcia-Marques et al. (2010) proposed that the implicit theory of personality corresponds to a specialized associative memory structure. Garcia-Marques and colleagues used an adapted version of the Deese-Roediger-McDermott (DRM) paradigm (Deese, 1959; Roediger and McDermott, 1995) to provide evidence for a memory structure underlying impressions of personality that shares the same flexible advantages of other associative memory structures and, as a consequence, is also prone to the same performance costs, such as the occurrence of specific and predictable patterns of impression formation false memories. We now argue that some of the patterns of impression formation false memories form the basis of classical impression formation effects such as the centrality effect (Asch, 1946).

### The implicit theory of personality as an associative memory structure

Bruner and Tagiuri (1954) argued that when social perceivers form impressions of a target's personality, they are guided by systematic expectancies about which traits tend to go together in a given personality. Using techniques such as multidimensional scaling, Rosenberg et al. (1968) were able to identify which personality traits cluster together, and proposed that the implicit theory of personality corresponds to a semantic space defined by two evaluative dimensions—an intellectual dimension that maps intellectual positive and negative personality traits; and a social dimension that maps social positive and negative traits. Such semantic structure would allow social perceivers to go beyond the information given and generate missing information when forming impressions. That is, when social perceivers form impressions of a target, the available target information (e.g., traits) is used to infer other traits that occupy neighboring positions in the implicit theory of the personality semantic space, filling in for missing information and allowing for a more comprehensive view of the target's personality.

Recently, and following the footsteps of Rosenberg et al. (1968), we have showed that the implicit theory of personality can be seen as an associative memory structure organized according to the implicit beliefs people have about which personality traits usually co-occur in the same person (Garcia-Marques et al., 2010). The flexibility of associative memory structures has been systematically studied, using the DRM paradigm. In the DRM (Deese, 1959; Roediger and McDermott, 1995), participants are presented with a list of the words most commonly associated with a critical non-presented word (e.g., the words *sour, candy, sugar*, or *bitter*, are all associated to the critical word *sweet*) and when they are later asked to recall or recognize the presented words, participants very often intrude or falsely recognize the critical non-presented word (in the example, *sweet*). Following the idea that the implicit theory of personality can be conceived as an associative memory structure, we have shown that presenting personality traits that load more heavily on a given positive or negative cluster of a personality dimension leads to false recognition of non-presented personality traits that occupy the central positions in the cluster of those presented traits (Garcia-Marques et al., 2010). It was argued that false recognition of these traits is due to the activation that spreads from the traits presented at study to non-presented traits that occupy the most central positions in the congruent cluster. Moreover, the level of false recognition of associated personality traits was much higher when participants were asked to form an impression about someone's personality described by the presented words than when participants were asked to simply memorize the presented words. This finding suggests not only that the implicit theory of personality works as an associative memory structure but also that it is a highly specialized semantic space, recruited when people are forming impressions of personality and thus conditional to that processing goal.

### The centrality effect and associative memory structures

In the following studies, we propose studying the memory basis of the classic centrality effect in impression formation (Asch, 1946). According to Asch the extent and direction to which one goes beyond the initial information provided by a given trait is variable and some traits carry much more weight than others in the final impression of personality. Specifically, in a set of studies in which the participants formed impressions of a target's personality from a list of several traits that supposedly described the target, Asch (1946) showed that the mere replacement of one of the traits by its opposite (e.g., warm vs. cold) affected the participants' impressions. Asch (1946) called these traits “central traits.” The traits that had the smallest impact on the impression formed were called “peripheral traits.” Wishner (1960), on the other hand showed that trait centrality is a function of its novelty in the context of other available traits. In addition, Rosenberg et al. (1968) showed that centrality was in great part a matter of dimensional novelty. That is, when people form an impression of personality, they attempt to place the target in the bi-dimensional semantic space that underlies impressions (Rosenberg et al., 1968). To do this, social perceivers need information from both dimensions. When one dimension is well represented by the majority of the available traits, and the other dimension is represented by a single trait, this single trait will gain a disproportionate inferential weight. Thus, the centrality effect can be seen as a consequence of the nature of impression formation. As aforementioned, the implicit theory of personality may be conceived as an associative memory structure. Following this line of reasoning, we argue that the inferential activity triggered by just one trait as long as it is important or novel enough, may be sufficient to produce a predictable pattern of false memories. If we consider that central traits play a crucial role in impression formation, we can assume that activation of a critical non-presented trait can occur not only by convergent spreading activation processes, but also by active inference processes. We use the term “active inference” to distinguish it from the passive accumulation of activation that spreads from semantic associates as it is supposed to happen in the original form of the DRM paradigm (Roediger and McDermott, 1995). In the case of centrality effects, the presence of a single trait should affect a number of traits in the same dimension suggesting that the underlying process is different in nature from passive and cumulative spreading activation that requires the activation via presentation of several traits that jointly spread to the critical one. Instead, centrality effects seem to be the result of active inferential processing implied in impression formation, that requires the activation via presentation of only one trait and that activation causes the activation of several traits, thus unlikely by spreading activation that accumulates in a critical word.

By using an adaptation of the DRM paradigm and creating lists that include a central trait it was possible to check how the presence of this single trait influences the false recognition of other non-presented personality traits. In two of the following experiments (Experiments 2 and 3) we used the same procedure used by Garcia-Marques et al. (2010) except that the study lists included not the central trait of the cluster represented in the list, but a central trait of the opposite dimension (with congruent or incongruent valence). For example, in the social positive list, the social positive central trait was replaced by an intellectual central trait, that was either positive or negative, depending on the condition. We predict that participants will produce false memories from the same dimension and valence of the list (for example, the social positive list will result in the false recognition of non-presented social positive traits). More importantly, we predict that participants will produce false memories from the central trait dimension congruent with its valence because this single central trait will play a major role in the impression formed. However, if these false memories congruent with the central trait dimension and valence only occur when we instruct participants to form impressions of personality, the assumption that the implicit theory of personality is a semantic space conditional to the impression formation will receive support. If such false memories occur, they will be another hallmark of the activation of a dedicated memory structure underlying impressions of personality.

However, before we set out to test the interplay between memory processes and the centrality effect using the developed materials, we did two preliminary studies (Experiments 1A and 1B) to make sure that in a simple impression formation task those materials would elicit the centrality effect as described by Asch (1946). Experiment 1A addressed the role of what we considered central traits and Experiment 1B addressed the role of what we considered peripheral traits, thus we expect to find a centrality effect in experiment 1A and no effects in Experiment 1B. Following Asch's (1946) procedure, after encoding under impression formation settings a list of personality traits (social or intellectual) plus a central trait of the opposite dimension (with congruent or incongruent valence), participants in Experiment 1A were simply asked to select traits from a check list that were congruent with the impression they formed. Based on Asch's (1946) results, we predicted a centrality effect. That is, the acceptance level of traits from the dimension of the central trait should be higher for traits with the same valence than from traits with the opposite valence. Experiment 1B replicated Experiment 1A's procedure except that central traits were replaced by peripheral traits. As shown by Asch (1946, Experiment 3; see also Rosenberg et al., 1968) peripheral traits provide low dimensional novelty and thus should not lead to substantial changes in the impressions of personality formed by the participants and therefore, we predict no valence-based differences between traits acceptance in Experiment 1B. Thus, the use of peripheral traits in Experiment 1B should allow us to show that the abovementioned centrality effect is in fact specific to central traits and not merely the result of adding traits of the opposite dimension of the lists. This will be important to justify the selection of a given set of central traits in Experiments 2 and 3.

If we replicate Asch (1946)'s original results (Experiments 1A and 1B) and if we find evidence of the centrality effect in a DRM like memory paradigm (Experiments 2 and 3), we would be gathering evidence supporting our hypothesis that centrality effects in impression formation can be explained by the representation of the implicit theory of personality in memory.

## Experiment 1A

In Experiment 1 we set out to replicate Asch (1946) centrality effect. The procedure used in this experiment was similar to Asch's procedure—we presented personality traits describing a target person and then gave participants a checklist of personality traits and asked them to select the ones compatible with the impression they formed. But, differently from Asch, we used descriptions built in a way to reflect the implicit theory of personality. Also, we predict the patterns of trait acceptance based on the central trait we added to the description. This added central trait was from a different dimension of the remaining traits presented in the description. We expect that the acceptance of traits of the same valence and dimension would be higher than the acceptance of traits with opposite valence but of the same dimension of the central trait, independently of the valence of the other traits included in the list (always from a different dimension of the central trait). This was essentially the results pattern that Asch obtained. The goals of this experiment were to replicate the centrality effect in an impression formation paradigm, and to establish the pattern of false memories that we expect to obtain in subsequent experiments.

### Methods

#### Participants

Seventy-three undergraduate students from University of Lisbon participated in exchange for course credit.

#### Materials

We used the same lists that Garcia-Marques et al. (2010) used to study the effect of false memories within the implicit theory of personality. After a multidimensional scaling and a 4-Way Cluster Analysis of the traits most used to describe people, four clusters of personality traits were identified (social positive, social negative, intellectual positive, and intellectual negative) and the traits in each one were organized in terms of their distance to the central trait. For each cluster, the central trait is the trait that loads more heavily in the defining characteristics of the cluster. The distance between each trait and the central trait are assumed to reflect its association strength within the implicit theory of personality. From each cluster, the five words closest to the central trait were never presented. The following nine traits in each cluster were used to form the lists. For each list we added the central trait from the opposite dimension, with the same or opposite valence of the list. We also added six athematic words (i.e., words that were not personality traits but objects instead), to maintain the same composition of the lists used in previous experiments (Garcia-Marques et al., 2010). In total, we obtained eight lists with 16 words each—social positive list with intellectual central trait (positive or negative); social negative list with intellectual central trait (positive or negative); intellectual positive list with social central trait (positive or negative); intellectual negative list with social central trait (positive or negative). The order of presentation of the words was the same for all participants. The central trait was always presented in the middle of the list and the other nine traits were presented in ascending order of distance (starting with the trait closest to the centroid).

From each cluster, the five words closest to the central trait were never presented and were used to form a checklist composed of 20 non-presented critical traits. Please see Supplementary Material for the translation of the complete list of traits used.

#### Design

The design was 2 dimension of the list (social vs. intellectual) × 2 valence of the list (positive vs. negative) × 2 valence of the central trait from the opposite dimension (positive vs. negative), with all factors between participants. The dependent variables measured by a checklist procedure were the proportion of accepted critical traits of the same valence minus the equivalent proportion of trait of opposite valence (both from the same dimension of the list) and the same difference in proportions for the accepted critical traits of the central trait dimension.

#### Procedure

The experiment was conducted in small groups of 2–10 participants, randomly assigned to each one of the eight lists. All participants were asked to form an impression of personality of a target person described by a list of words allegedly provided by people who were well acquainted with the target. Participants were alerted to the fact that the words used to describe the target could be adjectives or nouns and were given an example. After participants read the instructions, one of the eight lists was auditorily presented. After the list presentation, the experimenter gave the participants a checklist with 20 personality traits with instructions to select the ones that could belong to the description of the person they just heard, i.e., the traits that were congruent with the description of the person. After the experiment participants were thanked and fully debriefed.

### Results

The mean acceptance of the different types of traits given the valence and dimension of the list and of the central trait are presented in Table 1.

**Table 1:** Proportion of accepted critical traits as a function of list and central trait dimension and valence in Experiment 1A (standard deviations are in parentheses).

	**Intellectual − critical traits**	**Intellectual + critical traits**	**Social − critical traits**	**Social + critical traits**
Social − List/Intellectual − CT	0.22 (0.31)	0.37 (0.35)	0.70 (0.39)	0.15 (0.28)
Intellectual − List/Social − CT	0.67 (0.36)	0.09 0.15)	0.18 (0.21)	0.16 (0.19)
Social − List/Intellectual + CT	0.11 (0.23)	0.47 (0.32)	0.69 (0.32)	0.00 (0.00)
Intellectual − List/Social + CT	0.60 (0.27)	0.04 (0.08)	0.00 (0.00)	0.52 (0.30)
Social + List/Intellectual − CT	0.09 (0.15)	0.29 (0.36)	0.18 (0.35)	0.62 (0.41)
Intellectual + List/Social − CT	0.00 (0.00)	0.78 (0.19)	0.16 (0.22)	0.02 (0.07)
Social + List/Intellectual + CT	0.08 (0.19)	0.74 (0.28)	0.10 (0.25)	0.82 (0.30)
Intellectual + List/Social + CT	0.02 (0.07)	0.87 (0.14)	0.02 (0.07)	0.42 (0.31)

To test the impact of the list in the acceptance of critical non-presented traits of the same dimension, a 2 list dimension (social vs. intellectual) × 2 central trait valence (positive vs. negative) × 2 list valence (positive vs. negative) ANOVA was performed on the difference between the proportions of positive and negative traits of the same dimension of the list. The only effect to emerge was a list valence main effect, *F*_(1, 65)_ = 103.99, *p* < 0.001, η^2^ = 0.60, indicating that the difference was positive when the list valence was positive (*M* = 0.68, *SD* = 0.49) and negative when the list valence was negative (*M* = −0.53, *SD* = 0.52).

To test the impact of the central trait in the acceptance of critical non-presented traits of the same dimension, a 2 list dimension (social vs. intellectual) × 2 central trait valence (positive vs. negative) × 2 list valence (positive vs. negative) ANOVA was performed on the difference between the proportions of positive and negative traits of the same dimension of the central trait. The only effect to emerge was a central trait valence main effect, *F*_(1, 65)_ = 14.44, *p* = 0.001, η^2^ = 0.17, indicating that the difference was more positive when the central trait was positive (*M* = 0.43, *SD* = 0.46) than when it was negative (*M* = 0.05, *SD* = 0.39).

### Discussion

These results replicate Asch (1946) by showing that, when describing a target, replacing a single trait by a trait with opposite valence has a profound impact in the inferences made about the target. As shown by Asch (1946), inferences regarding traits of the same valence and dimension of the list are not affected by this manipulation of the central trait. It is also worth mentioning that inferences regarding the central trait dimension were more readily made when the central trait was positive than when it was negative (the difference is positive in the first case and near zero in the second case). The same asymmetry could be found in Asch's results given that the inclusion of the trait “warm” led to more consensual inferences regarding traits of the same dimension and valence than the inclusion of “cold.” For instance, when “warm” was included in the list 90% of the participants thought the target was “happy” and 94% thought the target was “good natured.” But when “warm” was replaced by “cold,” these percentages fell to 66 and 83%, respectively (Asch, 1946, Experiment 1; see also Hamilton et al., 2009). This “positivity effect” is also in line with a recent study that showed stronger halo effects for positive information than for negative information and argued that it was due to more similarities between positive information than negative information, which should allow for not only trait to trait inferences but also general impression to trait inferences (Gräf and Unkelbach, 2016). Similarly, Bruckmüller and Abele (2013) also found that negative personality information tends to be less clustered and thus less similar than positive personality information. These novel differences in similarity between positive and negative traits might contribute to explain this positivity effect we obtained.

Given the obtained results, the traits we chose as central seem able to elicit the same centrality effect described by Asch (1946) in an impression formation paradigm. However, we cannot discard the possibility that any trait from a different valence than the list's traits would have the same effect and, if that was the case, this effect would not be unique to central traits. The following experiment tests this idea by adding traits that are also from different valences relatively to the ones in the list but that are peripheral given the implicit theory of personality we mapped.

## Experiment 1B

Experiment 1B closely replicated Experiment 1A procedure and used the same materials except that the central traits previously used were replaced by peripheral traits. Given the low informativeness of peripheral traits (Rosenberg et al., 1968), we predict that, independently of the valence of the other traits included in the list (always from a different dimension of the peripheral trait), the acceptance of traits of the same valence and dimension of peripheral traits would not be substantially higher than the acceptance of traits with opposite valence but of the same dimension of the peripheral traits. In other words, in contrast to Experiment 1A results, we do not expect to obtian a centrality effect, thus showing the crucial difference between central and peripheral traits.

### Methods

#### Participants

Eighty undergraduate students from University of Lisbon participated in this study for course credit.

#### Materials

The same lists of traits of Experiment 1A were used except that central traits were replaced by peripheral traits. The 4-Way Cluster Analysis of the traits (Garcia-Marques et al., 2010), mentioned in Experiment 1A, was used to identify the peripheral trait for each cluster (social positive, social negative, intellectual positive, and intellectual negative). Rather than choosing the traits farther away from each centroid we consider the global amplitude of the traits' unscaled squared euclidian distances from their centroids (varying between 2.07 and 3.69, see Ferreira et al., 2011) and define *peripheral traits* as those which had a fixed squared euclidean distance of 3 (or more) from the respective centroid. This arbitrary criterion was defined to guarantee that the peripheral traits' weak associations with the centroids were kept constant across clusters. Moreover, by implementing this criterion instead of using the traits farther away from each centroid we provide a more conservative test of the hypothesis concerning the differential impact of central and peripheral traits in impression formation since we make sure that the peripheral traits are still well within each cluster. Please see Supplementary Material for the translation of the complete list of traits used.

#### Design and procedure

Experiment 1B used the same design and procedure as in Experiment 1A.

### Results

The mean acceptance of the different types of traits given the valence and dimension of the list and of the peripheral trait are presented in Table 2.

**Table 2:** Proportion of accepted critical traits as a function of list and peripheral trait (PT) dimension and valence in Experiment 1B (standard deviations are in parentheses).

	**Intellectual − critical traits**	**Intellectual + critical traits**	**Social − critical traits**	**Social + critical traits**
Social − List/Intellectual − PT	0.12 (0.06)	0.34 (0.07)	0.86 (0.05)	0.00 (0.06)
Intellectual − List/Social − PT	0.66 (0.06)	0.10 (0.07)	0.24 (0.05)	0.12 (0.07)
Social − List/Intellectual + PT	0.28 (0.06)	0.30 (0.07)	0.86 (0.05)	0.00 (0.06)
Intellectual − List/Social + PT	0.54 (0.06)	0.10 (0.07)	0.26 (0.05)	0.10 (0.06)
Social + List/Intellectual − PT	0.00 (0.06)	0.46 (0.07)	0.00 (0.05)	0.86 (0.06)
Intellectual + List/Social − PT	0.02 (0.06)	0.70 (0.07)	0.08 (0.05)	0.18 (0.06)
Social + List/Intellectual + PT	0.08 (0.06)	0.36 (0.07)	0.04 (0.05)	0.80 (0.06)
Intellectual + List/Social + PT	0.00 (0.06)	0.72 (0.07)	0.00 (0.05)	0.36 (0.06)

To test the impact of the list in the acceptance of critical non-presented traits of the same dimension of the list, a 2 list dimension (social vs. intellectual) × 2 peripheral trait valence (positive vs. negative) × 2 list valence (positive vs. negative) ANOVA was performed on the difference between the positive and negative traits of the same dimension of the list. As expected, a list valence main effect emerged, *F*_(1, 72)_ = 407.54, *p* < 0.001, η^2^ = 0.67, indicating that the difference was positive (*M* = 0.76) when the list was positive and negative (*M* = −0.68) when the list was negative. This effect was qualified by a list valence X list dimension interaction, *F*_(1, 72)_ = 10.93, *p* = 0.001, η^2^ = 0.29, indicating that this difference between list valence was more pronounced for the social lists (*M* = −0.86 when the list was negative and *M* = 0.81 when the list was positive) when compared to the intellectual lists (*M* = −0.50 when the list was negative and *M* = 0.70 when the list was positive).

To test the impact of the peripheral trait in the acceptance of critical non-presented traits of the same dimension, a 2 list dimension (social vs. intellectual) × 2 peripheral trait valence (positive vs. negative) × 2 list valence (positive vs. negative) ANOVA was performed on the difference between the positive and negative traits of the same dimension of the peripheral trait (i.e., the same traits that were used in Experiment 1A). A list valence main effect emerged, *F*_(1, 72)_ = 69.49, *p* < 0.001, η^2^ = 0.85, indicating that the difference between the proportion of positive and negative traits was slightly positive (*M* = 0.19) when the list was negative; and negative (*M* = −0.54) when the list was positive. Note that this effect of list valence is independent of the effects of the peripheral traits and may reflect the strong effect of the valence of the trait dimension overrepresented (the list dimension) and a compensation in valence for the trait dimension underrepresented (the peripheral trait dimension) in the same trait list. That is, for instance, a positive intellectual list generated a very positive impression in the intellectual dimension but a negative impression in the social dimensions (Yzerbyt et al., 2008). This effect was qualified by a list valence by list dimension interaction, *F*_(1, 72)_ = 29.83, *p* < 001, η^2^ = 0.13 indicating that the aforementioned tendency to offset the list valence was stronger for the intellectual lists (*M* = 0.50 when the list was negative, and *M* = −0.70 when the list was positive) than for the social lists (*M* = −0.12 when the list was negative and *M* = −0.37 when the list was positive). More importantly, there was no peripheral trait valence main effect, *F* < 1, and this factor did not interact with any of the other factors (list valence and/or list dimension).

### Discussion

Taken together with Experiment 1A, the current results further replicate Asch (1946) by showing that, when describing a target, replacing a single trait by a trait with opposite valence only impacts the inferences made about the target if this trait is a central trait (i.e., highly informative of the dimension at stake). In fact, by replacing the central traits used in Experiment 1A by peripheral traits we made the centrality effect disappear in the same manner as Asch did (see Asch, 1946; Experiment 3).

We also found a tendency to compensate the valence of the trait dimension over-represented in the list (the list dimension) with the valence of the accepted traits of the under-represented trait dimension (the peripheral trait dimension), particularly in the case of the intellectual lists, which may be seen as an instance of the so called compensation effect (Judd et al., 2005), and does not replicate the positivity effects mentioned earlier (Bruckmüller and Abele, 2013; Gräf and Unkelbach, 2016), possibly due to a decrease in overall symmetry, which would be consistent with the dynamic nature of the associations between personality traits that we addressed in our previous studies (Garcia-Marques et al., 2010).

Summing up, this experiment jointly with Experiment 1A showed that our materials seem able to elicit the same centrality effect described by Asch (1946) in an impression formation paradigm. The next two experiments were designed to test if we could extend the same effect with a false memory paradigm. Given that we showed a centrality effect with the traits used in Experiment 1A and a lack of effect with the traits used in Experiment 1B, Experiments 2 and 3 will only use central traits as we wouldn't predict an effect of peripheral traits and adding that comparison would double the number of conditions in each experiment, decreasing the power.

## Experiment 2

In Experiment 2 we used the same materials of Experiments 1A and 1B. Our goal was to replicate the centrality effect initially described by Asch (1946) using an adaptation of a memory paradigm (the DRM; Roediger and McDermott, 1995). We expect to conceptually replicate the centrality effect obtained in Experiment 1A, this time translated into false alarms in a recognition test. We thus predict an increase of false recognitions of critical words from the same dimension of the central trait. Moreover, given the role that valence played in Experiment 1A, participants are expected to make more false recognitions matching the valence and dimension of the central trait when it is positive than when it is negative. This results pattern should only occur when participants actively form impressions of personality and not when participants just memorize the target's description.

### Methods

#### Participants

One power analysis using the Gpower computer program (Faul et al., 2007) indicated that a total sample of 79 people would be needed to detect an effect as large as the one obtained in Experiment 1A for the central trait manipulation (η^2^ = 0.17) with 80% power using an ANOVA with 3 independent variables, two levels each, and a total of 6 groups, at 0.05. Please note that we used the smallest effect size obtained in Experiment 1A. We almost doubled the estimated sample needed because of credit availability for students and to ensure that despite differences in design we would still have power to detect an effect. Thus, one hundred and forty-nine undergraduate students from University of Lisbon participated in exchange for course credit.

#### Materials

We used the same lists of personality traits and athematic words used in Experiment 1A. But, instead of the checklist, we created a recognition test. The recognition test consisted of 42 words: five presented traits (from the 9 originally presented); 4 central traits (only one has been presented); the 20 non-presented critical traits (five from each cluster); 3 presented athematic words; 6 non-presented athematic words associated with the presented ones, 4 non-presented and non-associated athematic words. Thus, except for the five presented traits, the tests were equal for every participant. The sequence of presentation of the words was randomized for each subject. See Supplementary Material for the complete list of traits used in each list and in the recognition test.

#### Design

The design was a 2 encoding goals (memory vs. impression formation) × 2 dimension of the list (social vs. intellectual) × 2 valence of the list (positive vs. negative) × 2 valence of the central trait from the opposite dimension (positive vs. negative), with all factors between participants. The dependent variables measured in a recognition test were veridical recognition of the presented words included in the test and false recognition of non-presented words. To further analyze false recognition we used the difference between the proportion of positive and negative traits from the same dimension of the list or from the same dimension of the central trait.

#### Procedure

The experiment was conducted in small groups of 2–10 participants, randomly assigned to each one of the conditions (memory vs. impression formation) and one of the eight lists. Participants in the impression formation condition were asked to form an impression of personality of a target person described by a list of words allegedly provided by people who were well acquainted with the target, whereas participants in the memory condition were simply asked to memorize a set of words. Participants in the impression formation condition were alerted to the fact that the words used to describe the target could be adjectives or nouns and were given an example. After participants read the instructions, one of the eight lists was auditorily presented. After the list presentation, participants in the impression formation condition were given 90 s to “mentally revise the impression they formed about the target” and participants in the memory condition were given the same time to “rehearse the presented words as a preparation for an upcoming memory test.” After this study phase, all participants performed a distracter task for 10 min. The distracter task used was part of a different research project called “what's next” where participants were instructed to guess the outcomes on a series of coin tosses (head or tail). After the distracter, all participants performed a self-paced recognition test (see Supplementary Material for a translation of the verbatim instructions). After the experiment participants were thanked and fully debriefed.

### Results

As we did not predict pattern differences between social and intellectual traits (see Garcia-Marques et al., 2010; and Experiment 1A), the results were collapsed across dimension of the list (social and intellectual dimension) and dimension of the central trait (social and intellectual central trait)^1^. By collapsing across dimensions we ended up with four types of list: positive list with positive central trait, negative list with negative central trait, positive list with negative central trait, and negative list with positive central trait. Multi-factorial ANOVAs to test the effects of the independent variables 2 encoding goals (memory vs. impression formation) × 2 valence of the list (positive vs. negative) × 2 valence of the central trait from the opposite dimension (positive vs. negative) on each dependent variable were performed. All data are presented in terms of proportions. The frequencies from which proportions were calculated for each item type are as follows. For veridical recognition: list traits out of 5 (the number of traits presented in the study list and in the recognition test); the added central trait out of 1 (the number of central traits added to the study list and present in the recognition test); athematic words out of 3 (the number of athematic words presented in the study list and in the recognition test).

To analyze false recognition we used the difference between the proportion of positive and negative traits from the same dimension. We chose this procedure for two reasons (i) previous research has shown that there is a strong asymmetry in false recognition of positive and negative items (Garcia-Marques et al., 2010; Alves et al., 2015) and (ii) Asch (1946) studied the centrality effect using a checklist of polar opposites asking participants to convey their impressions of the target by choosing one of the opposites in each pair. We conceptually replicate this feature of Asch's original work by using the difference between the proportion of positive and negative traits from the same dimension. Thus, we computed the difference between the proportion of positive and negative traits falsely recognized from the same dimension of the list (each proportion was calculated out of 5, i.e., the number of critical words in the recognition test from each of the four combinations dimension-valence). We also computed the difference between the proportion of positive and negative traits falsely recognized from the dimension of the central trait (again, each proportion was calculated out of 5, i.e., the number of critical words in the recognition test from each of the four combinations dimension-valence). Finally, for athematic associated words, we computed proportions of falsely recognized items out of 6 (the number of non-presented athematic associates in the recognition test), and for non-associated athematic words we computed proportions of falsely recognized items out of 4 (the number of non-presented, non-associated athematic words in the recognition test).

The proportions of veridical and false recognition for each dependent measure are presented in Table 3. Only comparisons that reached significance or directly relevant to our hypothesis are presented, and all the comparisons not presented did not reach significance. The level of correct recognition of presented traits did not differ between conditions, *F* < 1, with participants in the memory condition correctly recognizing 86% (*SD* = 16.54%) of the presented traits and participants in the impression formation condition correctly recognizing 87% (*SD* = 11.18%) of the presented traits. However, participants in the impression formation condition correctly recognized the added central trait more often than participants in the memory condition (*M* = 0.85, *SD* = 0.45 vs. *M* = 0.71, *SD* = 0.42), *F*_(1, 133)_ = 4.33, *p* = 0.039, MSE = 0.17, η^2^ = 0.03. Also the correct recognition of the presented athematic words differed between conditions, *F*_(1, 133)_ = 4.00, *p* = 0.048, MSE = 0.05, η^2^ = 0.03 with participants in the impression formation condition correctly recognizing more athematic words than participants in the memory condition (*M* = 0.86, *SD* = 0.27 vs. *M* = 0.78, *SD* = 0.25). The false recognition of athematic words associated to the presented athematic words did not differ between conditions, (*M* = 0.10, *SD* = 0.18 vs. *M* = 0.12, *SD* = 0.17, for participants in the impression formation condition and participants in the memory condition, respectively), *F* < 1. In any case, the level of false recognition of athematic words associated to the presented athematic words was significantly different from the level of false recognition of athematic words that were not associated to the presented athematic words (*M* = 0.11, *SD* = 0.12 vs. *M* = 0.04, *SD* = 0.12, for associated athematic words and non-associated athematic words, respectively), *F*_(1, 133)_ = 23.18, *p* < 0.001, MSE = 0.01, η^2^ = 0.15.

**Table 3:** Proportion of trait hits, central trait hits, athematic hits; and differences between proportions of false memories of opposite valence (for list and central traits) by list valence and central trait valence (intellectual and social dimensions were aggregated), for the recognition test in Experiment 2.

	**Presented list**			
	**Positive list**	**Positive list**	**Negative list**	**Negative list**
	**Positive centroid**	**Negative centroid**	**Positive centroid**	**Negative centroid**
**IMPRESSION FORMATION**				
**Hits**				
Trait words (List)	0.86 (0.23)	0.92 (0.12)	0.89 (0.17)	0.84 (0.20)
Centroid	0.90 (0.31)	0.95 (0.22)	0.80 (0.41)	0.75 (0.44)
Athematic words	0.83 (0.23)	0.85 (0.17)	0.88 (0.19)	0.85 (0.23)
**False alarms**				
Difference between positive and negative list clusters	0.44 (0.23)	0.59 (0.22)	−0.35 (0.28)	−0.43 (0.26)
Difference between positive and negative centroid clusters	0.20 (0.23)	0.21 (0.24)	0.07 (0.29)	−0.10 (0.15)
Athematic associates	0.12 (0.14)	0.08 (0.10)	0.11 (0.14)	0.09 (0.13)
**MEMORY**				
**Hits**				
Trait words (List)	0.84 (0.18)	0.92 (0.10)	0.86 (0.17)	0.84 (0.14)
Centroid	0.82 (0.39)	0.62 (0.50)	0.70 (0.47)	0.69 (0.48)
Athematic words	0.75 (0.25)	0.83 (0.27)	0.76 (0.23)	0.77 (0.14)
**False alarms**				
Difference between positive and negative list clusters	0.38 (0.33)	0.20 (0.24)	0.04 (0.33)	−0.11 (0.44)
Difference between positive and negative centroid clusters	0.08 (0.14)	0.22 (0.25)	0.08 (0.27)	−0.08 (0.22)
Athematic associates	0.07 (0.12)	0.08 (0.16)	0.19 (0.19)	0.12 (0.14)

*standard deviations are in parentheses*.

More interestingly, the difference between positive and negative false alarms from the same dimension of the list, i.e., difference between the proportions of false alarms for traits from the positive and negative list clusters, was influenced by the valence of the list, especially in the impression formation condition. The ANOVA showed a main effect of the valence of the list, *F*_(1, 133)_ = 168.35, *p* < 0.001, MSE = 0.09, η^2^ = 0.56. Positive lists tend to lead to a positive difference between the proportions of positive and negative false alarms from list's dimension (*M* = 0.40, *SD* = 0.26) and negative lists tend to lead to a negative difference between the proportions of positive and negative false alarms from list's dimension (*M* = −0.21, *SD* = 0.26). Nevertheless, there was an interaction between the valence of the list and the encoding goals condition, *F*_(1, 133)_ = 37.43, *p* < 0.001, MSE = 0.09, η^2^ = 0.22 such that the effect of list valence on the difference between the proportions of positive and negative false memories corresponding to the list dimensions was much more accentuated under impression formation (*M* = 0.52, *SD* = 0.32 vs. *M* = −0.39, *SD* = 0.32, for positive and negative lists, respectively) than under memory instructions (*M* = 0.29, *SD* = 0.29 vs. *M* = −0.04, *SD* = 0.30, for positive and negative lists, respectively).

For false recognition of traits from the central trait cluster, we were interested to see if the valence of the central trait would have an impact in the difference between the proportions of positive and negative false memories of traits from the central trait's dimension. Contrary to what we predicted, such main effect failed to occur, *F* < 1. Also, there was no interaction between the valence of the central trait and the encoding goal condition, *F* < 1. So, the impact of the central trait valence in the difference between the proportions of positive and negative false memories from the central trait dimension did not differ significantly for participants in impression formation conditions (*M* = 0.14, *SD* = 0.25 vs. *M* = 0.06, *SD* = 0.25, for positive and negative central traits, respectively) and memory conditions (*M* = 0.08, *SD* = 0.23 vs. *M* = 0.07, *SD* = 0.24 for positive and negative central traits, respectively).

### Discussion

In Experiment 2 we found that participants asked to form impressions of personality did not correctly recognize the presented personality traits more often than participants asked to simply memorize them. However, compared to memory participants, impression formation participants showed a greater impact of the nature of the presented traits, translated into greater false recognitions congruent with the dimension and valence of the presented traits.

Nevertheless, veridical recognition of the central trait was higher for participants forming an impression than for those memorizing the words. This result is reminiscent of the Von Restorff effect (Calkins, 1896; von Restorff, 1933) also referred to as isolation effect or distinctiveness effect (e.g., Hunt, 1995; Hunt and Lamb, 2001; Kelley and Nairne, 2001). The Von Restorff effect illustrates how people typically show better memory for a particular item that stands out in some way from similar items. It has been proposed that item's distinctiveness is likely to reduce proactive and retroactive interference (e.g., Underwood, 1964); to lead to deeper processing levels of encoding (e.g., Lockhart et al., 1976); and/or to produce more specific and unique memory cues (e.g., Tulving, 1983). Regardless of the relative contribution of these different accounts, the distinctiveness of the central trait here reported seems to be a direct result of the activation of a specific semantic space underlying the implicit theory of personality, which was triggered by impression formation instructions.

Thus, it seems that the exact same information was presented across conditions but encoded and represented differently under impression formation and memory settings. This probably occurred because for impression formation participants the added central trait brought unique information about its dimension whereas the other traits all belong to the same (opposite) dimension and thus they did not play a special role individually (Asch, 1946). In other words, distinctiveness of central traits is not the result of some invariant property of the presented information. These traits only become distinctive under adequate processing goals. Certainly, specific or *ad hoc* goals may lead to momentary distinctiveness of certain items. However, and against the prediction, we could not find an effect for the valence of the added central trait on the valence of false recognitions from its dimension. In hindsight, because of the high levels of veridical recognition of the added central trait for the impression formation condition, it could be argued that participants were using a disqualifying monitoring strategy similar to a recall to reject strategy (Gallo, 2004). This type of strategy would capitalize in the high distinctiveness of central traits in impression formation. In such case, even if impression formation instructions successfully activated non-presented traits semantically close to the presented central traits, participants may have been able to use the high distinctiveness of central traits to infer that no other or very little other information was presented regarding the central trait dimension, thus avoiding false recognitions of traits from that dimension. In sum, this result may indicate that the distinctive nature of central traits may have an important role in monitoring and preventing false memories (Schacter et al., 2001; Hege and Dodson, 2004).

## Experiment 3

Given the results of Experiment 2, we designed a third experiment to try to decrease the likelihood of participants using a disqualifying monitoring strategy to reject traits from the cluster of the central trait (Gallo, 2004). More specifically, we added time pressure at test. According to Benjamin (2001), time pressure at the time of a recognition test increases the level of false alarms. We expect that, by making monitoring more difficult, the effects of the central trait will be more noticeable and more traits from the central trait cluster will be falsely recognized. However, this should only occur for the impression formation condition because it seems that participants in this condition were the ones using a disqualifying monitoring strategy in the first place, which would explain the higher level of veridical recognition of the added central trait for these participants when compared to memory condition participants. Thus, we expect to obtain a higher level of false alarms for traits sharing the central trait's dimension and valence, for participants in the impression formation condition.

### Methods

#### Participants

The results of the power analysis reported in Experiment 2 were used to calculate the sample size needed for this experiment. Again, we increased the suggested sample of 79 and thus, 113 undergraduate students from University of Lisbon participated in exchange for course credit.

#### Materials

The materials were the same used in Experiment 2.

#### Design

The design and dependent measures were the same of Experiment 2.

#### Procedure

The procedure was the same of Experiment 2, except that during the recognition test participants were tested under time pressure. Each word was presented on the screen for just 1 s, and participants had to give their response within that time (see Supplementary Material for a translation of the verbatim instructions). Whenever a subject was too slow in giving their response, a warning would come up on the screen, although their response was still considered for statistical analysis because: (i) we followed Benjamin (2001; Experiment 2) procedure; and (ii) failing to do so would probably result in a great loss of data, given the reduced number of trials used (i.e., words in the recognition test).

### Results

As in Experiment 2, for each dependent measure, multi-factorial ANOVAs were performed to test the effects of the independent variables 2 encoding goals (memory vs. impression formation) × 2 valence of the list (positive vs. negative) × 2 valence of the central trait (positive vs. negative)^2^. The proportions of veridical and false recognition for each dependent measure are presented in Table 4. Also as in Experiment 2, only comparisons that reached significance or directly relevant to our hypothesis are presented, and all the comparisons not presented did not reach significance. Correct recognition of presented traits did not differ between conditions, *F*_(1, 97)_ = 2.27, *p* = 0.135, MSE = 0.05, η^2^ = 0.02, with participants in the memory condition correctly recognizing 69% (*SD* = 21.69%) of the presented traits and participants in the impression formation condition correctly recognizing 75% (*SD* = 21.90%) of the presented traits. On the other hand, the added central trait was more often correctly recognized in the impression formation condition (*M* = 0.79, *SD* = 0.45) than in the memory condition (*M* = 0.61, *SD* = 0.45), *F*_(1, 97)_ = 4.47, *p* = 0.037, MSE = 0.20, η^2^ = 0.04. This result replicates the distinctiveness effect for the central traits under impression formation settings that was found in Experiment 2. On the contrary, correct recognition of the presented athematic words was higher for participants in the memory condition (*M* = 0.70, *SD* = 0.30) than for participants in the impression formation condition (*M* = 0.56, *SD* = 0.30), *F*_(1, 97)_ = 6.87, *p* = 0.010, MSE = 0.08, η^2^ = 0.07. However, the false recognition of athematic words associated to the presented athematic words did not differ between conditions, (*M* = 0.22, *SD* = 0.15 vs. *M* = 0.20, *SD* = 0.15, for participants in the impression formation condition and participants in the memory condition, respectively), *F* < 1. But the level of false recognition of athematic words associated to the presented athematic words was significantly different from the level of false recognition of athematic words non-presented and non-associated (*M* = 0.21, *SD* = 0.21 vs. *M* = 0.15, *SD* = 0.21, for associated athematic words and non-associated athematic words, respectively), *F*_(1, 97)_ = 8.97, *p* = 0.004, MSE = 0.02, η^2^ = 0.08.

**Table 4:** Proportion of trait hits, central trait hits, athematic hits; and differences between proportions of false memories of opposite valence (for list and central traits) by list valence and central trait valence (intellectual and social dimensions were aggregated), for the recognition test in Experiment 3.

	**Presented list**			
	**Positive list**	**Positive list**	**Negative list**	**Negative list**
	**Positive centroid**	**Negative centroid**	**Positive centroid**	**Negative centroid**
**IMPRESSION FORMATION**				
**Hits**				
Trait words (List)	0.86 (0.17)	0.69 (0.29)	0.77 (0.17)	0.68 (0.29)
Centroid	0.86 (0.36)	0.79 (0.43)	0.71 (0.47)	0.80 (0.41)
Athematic words	0.57 (0.24)	0.43 (0.38)	0.60 (0.23)	0.62 (0.25)
**False alarms**				
Difference between positive and negative list clusters	0.54 (0.25)	0.39 (0.23)	−0.49 (0.26)	−0.41 (0.40)
Difference between positive and negative centroid clusters	0.44 (0.27)	0.26 (0.37)	−0.06 (0.33)	−0.23 (0.21)
Athematic associates	0.26 (0.21)	0.17 (0.16)	0.21 (0.21)	0.22 (0.17)
**MEMORY**				
**Hits**				
Trait words (List)	0.71 (0.22)	0.79 (0.25)	0.61 (0.33)	0.64 (0.18)
Centroid	0.64 (0.50)	0.43 (0.51)	0.50 (0.52)	0.86 (0.36)
Athematic words	0.64 (0.33)	0.79 (21)	0.64 (0.31)	0.72 (0.26)
**False alarms**				
Difference between positive and negative list clusters	0.14 (0.31)	0.26 (0.24)	−0.16 (0.38)	−0.36 (0.44)
Difference between positive and negative centroid clusters	0.01 (0.30)	0.20 (0.25)	−0.03 (0.23)	−0.14 (0.23)
Athematic associates	0.23 (0.15)	0.18 (0.19)	0.16 (0.18)	0.24 (0.17)

*standard deviations are in parentheses*.

The results concerning the differences between the proportions of positive and negative false memories from the list dimension showed that we replicated the main effect of the list valence, and the interaction between list valence and condition, *F*_(1, 97)_ = 134.13, *p* < 0.001, MSE = 0.10, η^2^ = 0.58. Positive lists lead to a positive difference between the proportions of positive and negative false memories of traits from list dimension (*M* = 0.33, *SD* = 0.30) and negative lists lead to a negative difference between the proportions of positive and negative false memories of traits from list dimension (*M* = −0.36, *SD* = 0.30). The interaction between the valence of the list and the encoding goal also emerged, indicating that the impact of the valence of the list was more accentuated under impression formation goals (*M* = 0.46, *SD* = 0.32 vs. *M* = −0.46, *SD* = 0.32, for positive and negative lists, respectively) than under a memory encoding goal (*M* = 0.20, *SD* = 0.32 vs. *M* = −0.26, *SD* = 0.32, for positive and negative lists, respectively), *F*_(1, 97)_ = 15.14, *p* < 0.001, MSE = 0.10, η^2^ = 0.14. Regarding the false recognition of traits from the central trait cluster, there was no main effect of central trait valence in the magnitude of the difference between the proportions of positive and negative false memories of traits corresponding to the central trait dimension (*M* = 0.09, *SD* = 0.37 vs. *M* = 0.02, *SD* = 0.38, for positive and negative central traits, respectively), *F*_(1, 97)_ = 1.93, *p* = 0.168, MSE = 0.08, η^2^ = 0.02. Despite this, an interaction between the valence of the central trait valence and encoding goal did emerge, *F*_(1, 97)_ = 4.33, *p* = 0.040, MSE = 0.08, η^2^ = 0.04, such that the impact of central trait valence in the difference between the proportions of false memories of positive and negative traits corresponding to the central trait dimension was higher under impression formation (*M* = 0.19, *SD* = 0.26 and *M* = 0.01, *SD* = 0.27, for positive and negative central traits respectively) than under memory instructions (*M* = −0.01, *SD* = 0.26 and *M* = 0.03, *SD* = 0.26, for positive and negative central traits respectively).

### Discussion

Experiment 3 followed the same procedure of Experiment 2 differing only in the recognition test, where this time we asked participants to make a response within 1 s. This manipulation is supposed to decrease the possibility of a disqualifying monitoring strategy to operate (Gallo, 2004), such as “recall-to-reject,” and as a result increase the number of false alarms to the traits sharing the dimension and valence of the added central trait, especially under impression formation instructions. We replicated the results of Experiment 2, but also obtained the predicted effect of the valence of the added central trait, but only in the impression formation condition, indicating that the centrality effect is unique to impression formation goals.

The fact that we now obtained the predicted centrality effect, with the valence of the added central trait influencing the valence of the falsely recognized traits from the dimension of the central trait when participants were forming an impression of personality, supports the hypothesis that some traits are more important than others for impression formation. Namely, there are traits that bring unique information to an impression and that are most informative about a personality dimension because are closely connected to all the other traits highly saturated in that dimension (Asch, 1946; Wishner, 1960). It seems that, in some cases, the central trait may play an important role because it is highly recognizable but when time and cognitive resources are available, the fact that it is highly recognizable may hinder the false recognition of traits sharing the same characteristics via a disqualifying monitoring strategy, such as recall to reject (Gallo, 2004). In sum, the central trait seems to be distinctive and its inclusion prevents false alarms when there is enough time to make a response but, while forming impressions of personality and under time pressure, this false memory suppression is abolished, as suggested by Dodson and Hege (2005).

These results point to the importance of the organization of the personality traits in memory to the nature of the impressions formed. Since we obtained a centrality effect, only when participants performed an impression formation encoding task, we can assume that these effects are not taking place at retrieval. Instead, the obtained centrality effect seems to depend on a differential encoding of personality traits under impression formation that should reflect their representation in memory. Thus, we can reasonably argue that centrality effects in impression formation seem to reflect the organization of personality traits in memory.

## General discussion

In the two first experiments reported here we successfully replicated the centrality effect (Experiment 1A), as described by Asch (1946) and an absence of effect when peripheral traits were used (Experiment 1B). In Experiment 1A, we used lists of personality traits from a given dimension and valence, according to the implicit theory of personality, and added a central trait from the alternative dimension and with the same or opposite valence of the list. Participants were presented with one of these lists and asked to form an impression of the personality of a target person described by those traits. Later, participants were presented with a checklist and asked to select the traits compatible to the impression formed. We not only gathered support for the crucial role of central traits in impression formation but also the superiority of positive central traits when compared to negative traits, in terms of the inferential activity (number of inferences) they produce. The latter result is in line with the notion that the original “warm” “cold” effect proposed by Asch (1946) is mostly a “warm” effect (Hamilton et al., 2009). This superiority of positive traits might be due to a higher similarity between positive traits than between negative traits, as argued by Gräf and Unkelbach (2016), or to a closer clustering as Bruckmüller and Abele (2013) showed. These possibilities should be tested in future studies that combine the checklist procedure that Asch (1946) originally used and we replicated and similarity measures (e.g., Koch et al., 2016).

In Experiment 1B we used the same procedure and materials as in Experiment 1A, except that instead of adding a central trait we added a peripheral trait, i.e., a trait that still clearly belongs to a personality traits cluster but that is far from the centroid of its cluster. In Experiment 1B, as predicted, given Asch's (1946) work with peripheral traits, we did not obtain any effects attributable to the inclusion of the peripheral trait in the presented lists. Given these results, we hypothesized that in a memory task, if we presented lists of traits with central traits, an equivalent pattern of results should emerge in terms of false recognitions of traits belonging to the same dimension and valence of the added central trait. This should particularly occur when participants encode the target person's description under impression formation goals. We set out to test this idea in the following two experiments.

In Experiments 2 and 3 we obtained a higher impact of the list valence on the valence of the traits from the list dimension falsely recognized for participants forming impressions of personality than for participants who were instructed to memorize the same attributes. This effect was translated into a higher level of negative false alarms when the presented list was negative and a higher level of positive false alarms when the presented list was positive. On the other hand, there were no differences between conditions in the proportion of presented traits correctly recognized. Taken together, these results support previous studies and suggest that the implicit theory of personality functions as a specialized semantic structure which activation seems to be conditional to impression formation goals (Garcia-Marques et al., 2010). Hence these results are in agreement with the notion that social cognition may involve specialized brain structures and specific cognitive processes activated only when a social cognitive goal is operative (Mitchell et al., 2004).

More interestingly, in Experiment 3, we were able to find the centrality effect in false memories about personality traits, further replicating Experiment 1A. Our prediction was that, independently of the list valence, the valence of the added central trait would guide the false recognition of the personality traits from the dimension of the central trait, especially when participants were actively trying to form an impression of personality which implies positioning a target in the semantic space underlying the implicit theory of personality and actively inferring the traits that this target is likely to possess. In Experiment 2, we did not obtain this centrality effect. Instead we found an unexpected higher veridical recognition of the added central trait under impression formation goals than under memory goals. This may indicate that participants under impression formation instructions use the distinctiveness of the central trait to infer that no other or, at least, very few other traits from the same dimension were presented in the initial list describing the target's attributes. As a result, participants were able to reject these traits at test. This type of strategy has been dubbed a recall to reject strategy and it is a disqualifying monitoring strategy (Gallo, 2004). This is also consistent with a centrality effect (Asch, 1946) by which the central trait presented is easily recognizable because it is highly distinctive. Thus, this central trait possesses a type of secondary distinctiveness because its properties deviate from the properties of the other presented traits, by an inherent characteristic that is its position in the semantic space underlying the implicit theory of personality (Schmidt, 1991; Park et al., 2006) and by being the only one providing information about its dimension. Distinctiveness is known to increase veridical recognition (Hunt and McDaniel, 1993) and decrease false recognition of related lures (Schacter et al., 2001; Hege and Dodson, 2004). This increased distinctiveness could therefore facilitate the use of a disqualifying monitoring strategy at test (Gallo, 2010).

However, Dodson and Hege (2005) showed that the typical false recognition suppression due to item's distinctiveness occurs only when the recognition test is self-paced. Also, Benjamin (2001) found that false alarms increase when participants are tested under time pressure. This result has been attributed to a less effective monitoring under time pressure. Based on this previous research, in Experiment 3, we added a response dead line at test. By forcing participants to give their response rapidly, we were able to obtain the predicted effect of the valence of the central trait, but only for participants in the impression formation condition. Not only the added central trait was highly recognizable but also the level of false recognition of traits from the same cluster of the added traits was significantly higher than the level of false recognition of traits from the opposite cluster. As discussed above, this result is compatible with a high distinctiveness of the central trait—a secondary distinctiveness as Schmidt (1991) conceptualizes it. Thus, the personality traits referred to as central traits (Asch, 1946; Wishner, 1960) may simply be personality traits that are more distinctive from a memory point of view, given their intrinsic characteristics.

In addition, it seems that there is an evaluative consistency in our results that is supposed to reflect a basic principle of association between traits according to which positive traits are inferred from positive traits and vice-versa for negative traits (Heider, 1946; Bruner et al., 1958). The evaluative factor is so prevalent in human judgment (e.g., Osgood, 1962) that Brown (1965) considered it to be the simplest implicit personality theory. However, in our case, such ubiquitous implicit theory fails to account for the whole pattern of results. In fact, a mere principle of evaluative consistency cannot explain the higher impact of the central traits valence in the impression formation condition, regardless of the valence to the remaining traits of the list.

In sum, we successfully replicated the centrality effect in an impression formation task and, with the same materials, obtained false memories by using an adaptation of the DRM paradigm. Plus, we showed that the impact of the type of presented personality traits is larger when participants are under an impression formation goal than when participants are trying to memorize the presented words. Thus, our results support the claim that the implicit theory of personality is a conditional semantic network fully activated only under impression formation goals. One of the innovations in the reported experiments is that we tried to obtain false recognition of words associated with only one of the studied words. That is, we introduced a central personality trait and, taking into account the centrality effect reported by Asch (1946), we expected that this trait would be enough to promote false recognition of personality traits from its cluster. In fact, we obtained high levels of false recognition of personality traits stemming from these single traits, but only when participants were forming impressions of personality. To the best of our knowledge this is the first demonstration in DRM-like paradigm of false memories that derive from active inferential processing instead of processes of passive accumulation of convergent activation (e.g., Roediger and McDermott, 2000), in which instead of several items active due to their presentation spreading their activation to one common associate, one single item active due to presentation increases the false recognition of several other associated traits. Such results offer further support to the assumption that the semantic structure underlying the implicit theory of personality is conditional to the processing goal of forming an impression of personality. The fact that this effect was obtained only when participants were asked to make a fast recognition decision offers support to the hypothesis that a monitoring strategy may operate and prevent the impact of the central traits in impression formation in standard recognition tests.

Moreover, our results contribute to a clarification of the centrality effect in impression formation. The high levels of veridical recognition of the central trait added to the study list point to a distinctiveness approach to centrality. It seems that the centrality effect found in judgments about people's personality can be conceived as a distinctiveness memory effect, but that occurs only when that specific semantic memory structure that serves the impression of personality is active by a given processing goal. Thus, the distinctiveness effect is also conditional to the characteristics of the words within a given semantic memory space, activated according to specific processing goals. For further support of this distinctiveness based centrality, future studies should consider other possible ways to conceptualize the implicit theory of personality (e.g., Abele and Wojciszke, 2014). These alternative ways to organize personality traits in memory are also likely to yield false memories that mirror classic impression formation effects such as the centrality effect described here and provide further tests of the distinctiveness approach to trait centrality.

## Conclusion

In a series of experiments, we extended the DRM paradigm (Roediger and McDermott, 1995) to study the classic centrality effect in impression formation (Asch, 1946). In two checklist Experiments (1A and 1B), the effects obtained by Asch were replicated, indicating that the traits classified as central had more weight in the impression formed than the traits classified as peripheral. In Experiment 2, the processing goal was manipulated and the results pointed to a better recognition of the central trait included in a list when participants were instructed to form impressions of personality than when subjects were instructed to memorize the same list. This result points to a distinctiveness of the central trait that might contribute to the use of a recall-to-reject strategy at retrieval, preventing false alarms related to the central trait (Gallo, 2004). One way to avoid the use of a recall-to-reject strategy is introducing time pressure during retrieval Benjamin (2001). This is exactly what was done in Experiment 3 and, as predicted, the central traits were no longer better recognized than the other presented traits but there were more false recognitions of non-presented traits from the same valence and dimension of the central trait. Moreover, consistently across experiments, there was an advantage for positive traits over negative traits, providing evidence for a “warm” effect (Hamilton et al., 2009) and in accordance with the idea that there is a higher similarity between positive traits than between negative traits (Gräf and Unkelbach, 2016).

In sum, we successfully replicated the centrality effect in an impression formation task and, with the same materials, obtained false memories by using an adaptation of the DRM paradigm. Plus, we showed that the impact of the type of presented personality traits is larger when participants are under an impression formation goal than when participants are trying to memorize the presented words. Thus, our results support the claim that the implicit theory of personality is a conditional semantic network fully activated only under impression formation goals (Garcia-Marques et al., 2010). Hence these results are in agreement with the notion that social cognition may involve specialized brain structures and specific cognitive processes activated only when a social cognitive goal is operative (Mitchell et al., 2004).

## Ethics statement

This study was carried out in accordance with the recommendations of the Universidade de Lisboa, Faculdade de Psicologia, General directions for research with human participants, with written informed consent from all participants. All participants gave written informed consent in accordance with the Declaration of Helsinki. The protocol was approved by the research area of Faculdade de Psicologia, Universidade de Lisboa.

## Author contributions

LN and LG were responsible for the design, analysis, and write-up. MF collaborated on the write-up. TR collaborated on the data collection, with LN, and on the write-up. All authors collaborated on data interpretation.

### Conflict of interest statement

The authors declare that the research was conducted in the absence of any commercial or financial relationships that could be construed as a potential conflict of interest.

## Footnotes

^1^However, we ran ANOVAs, including list dimension as an independent variable. The results are presented in this footnote. For correct recognition of the presented traits, an interaction between processing goal and list dimension emerged *F*_(1, 133)_ = 4.96, *p* = 0.028, MSE = 0.14, η^2^ = 0.03, with participants in the impression formation condition correctly recognizing more social traits than intellectual traits (*M* = 0.92, *SD* = 0.17 vs. *M* = 0.83, *SD* = 0.17, respectively) but participants in the memory condition recognizing more intellectual traits than social traits (*M* = 0.88, *SD* = 0.17 vs. *M* = 0.85, *SD* = 0.17, respectively). For the difference between positive and negative false alarms from the same dimension of the list the ANOVA showed a main effect of list dimension, *F*_(1, 133)_ = 11.57, *p* < 0.001, MSE = 0.96, h^2^ = 0.03, qualified by an interaction between list dimension and processing goal, *F*_(1, 133)_ = 7.09, *p* < 0.001, MSE = 0.59, η^2^ = 0.02 (*M* = 0.08, *SD* = 0.29 vs. *M* = 0.05, *SD* = 0.29, for intellectual and social lists in the impression formation condition, respectively; and *M* = 0.27, *SD* = 0.29 vs. *M* = 0.02, *SD* = 0.29, for intellectual and social lists in the memory condition, respectively). Finally, for the difference between positive and negative false alarms from the same dimension of the central trait only a significant interaction between list dimension and processing goal emerged, *F*_(1, 133)_ = 5.78, *p* = 0.018, MSE = 0.30, η^2^ = 0.03 (*M* = 0.04, *SD* = 0.23 vs. *M* = 0.16, *SD* = 0.23, for intellectual and social lists in the impression formation condition, respectively; and *M* = 0.11, *SD* = 0.23 vs. *M* = 0.05, *SD* = 0.23, for intellectual and social lists in the memory condition, respectively). No other results reached significance.

^2^As in Experiment 2, we ran ANOVAs, including list dimension as an independent variable. The results are presented in this footnote. For correct recognition of the presented traits, a main effect of list dimension emerged *F*_(1, 97)_ = 18.87, *p* < 0.001, MSE = 0.92, η^2^ = 0.14, with participants recognizing more social traits than intellectual traits (*M* = 0.81, *SD* = 0.22 vs. *M* = 0.63, *SD* = 0.22, respectively). No other results reached significance.
