# Supplementary material for: Inferential Costs of Trait Centrality in Impression Formation: Organization in Memory and Misremembering
Source: Front Psychol. 2017 Aug 22;8:1408. doi: 10.3389/fpsyg.2017.01408 (PMC5572275; doi:10.3389/fpsyg.2017.01408)
Supplement: Supplementary file 1 [file DataSheet1.pdf]

APPENDIX 1

Presented lists and tests

Table 1. Translation of the lists of personality traits presented in Experiments 1A,1B, 2 and 3.

	**Same Valence**			
**LIST**	**Negative Social**	**Positive Intellectual**	**Positive Social**	**Negative Intellectual**
**CENTRALTRAIT**	**Negative Intellectual**	**Positive Social**	**Positive Intellectual**	**Negative Social**
1	*Intolerant*	*Smart*	*Sensitive*	*Unable*
2	Dishonest	Dinamyc	Friendly	Limited
3	Clock*	Clock*	Clock*	Clock*
4	*Mischievous*	*Efficient*	*Kind*	*Inattentive*
5	Count*	Count*	Count*	Count*
6	Rose*	Rose*	Rose*	Rose*
7	**<u>Irresponsible</u>**	**<u>Understanding</u>**	**<u>Cultured</u>**	**<u>Arrogant</u>**
8	Selfish	Quick	Reliable	Disorganized
9	Telephone*	Telephone*	Telephone*	Telephone*
10	*Cold*	*Creative*	*Sociable*	*Dependent*
11	Aggressive	Sharp	Joyful	Undecided
12	Blue*	Blue*	Blue*	Blue*
13	*Manipulative*	*Rational*	*Helpful*	*Dumb*
14	Egocentric	Independent	Honest	Customary
15	Cabinet*	Cabinet*	Cabinet*	Cabinet*
16	*Calculating*	*Fighter*	*Outgoing*	*Conservative*

	**Different Valence**			
**LIST**	**Negative Social**	**Positive Intellectual**	**Positive Social**	**Negative Intellectual**
**CENTRALTRAIT**	**Positive Intellectual**	**Negative Social**	**Negative Intellectual**	**Positive Social**
1	*Intolerant*	*Smart*	*Sensitive*	*Unable*
2	Dishonest	Dinamyc	Friendly	Limited
3	Clock*	Clock*	Clock*	Clock*
4	*Mischievous*	*Efficient*	*Kind*	*Inattentive*
5	Count*	Count*	Count*	Count*
6	Rose*	Rose*	Rose*	Rose*
7	**<u>Cultured</u>**	**<u>Arrogant</u>**	**<u>Irresponsible</u>**	**<u>Understanding</u>**
8	Selfish	Quick	Reliable	Disorganized
9	Telephone*	Telephone*	Telephone*	Telephone*
10	*Cold*	*Creative*	*Sociable*	*Dependent*
11	Aggressive	Sharp	Joyful	Undecided
12	Blue*	Blue*	Blue*	Blue*
13	*Manipulative*	*Rational*	*Helpful*	*Dumb*
14	Egocentric	Independent	Honest	Customary
15	Cabinet*	Cabinet*	Cabinet*	Cabinet*
16	*Calculating*	*Fighter*	*Outgoing*	*Conservative*

Note: Items marked with (*) are the athematic words added only to Experiments 2 and 3; traits in *italics* are the traits included in the recognition test; the bold and underlined traits are the central traits added to the list (from the opposite dimension), in Experiments 1A, 2, and 3. In Experiment 1B, the central traits Cultured, Arrogant, Irresponsible, and Understanding were replaced by Talkative, Conceited, Prudent, and Dependent, respectively.

Table 2. Constitution of the Recognition test used in Experiments 2 and 3.

WORD	TYPE	CLUSTER	OLD VS. NEW
Warm	Critical Trait	Social Positive	New
Giving	Critical Trait	Social Positive	New
Affectionate	Critical Trait	Social Positive	New
Fun	Critical Trait	Social Positive	New
Friend	Critical Trait	Social Positive	New
Envious	Critical Trait	Social Negative	New
Self-seeking	Critical Trait	Social Negative	New
Aloof	Critical Trait	Social Negative	New
Nasty	Critical Trait	Social Negative	New
Vengeful	Critical Trait	Social Negative	New
Skillful	Critical Trait	Intellectual Positive	New
Motivated	Critical Trait	Intellectual Positive	New
Organized	Critical Trait	Intellectual Positive	New
Determined	Critical Trait	Intellectual Positive	New
Competent	Critical Trait	Intellectual Positive	New
Unmotivated	Critical Trait	Intellectual Negative	New
Not-cultured	Critical Trait	Intellectual Negative	New
Incompetent	Critical Trait	Intellectual Negative	New
Slow	Critical Trait	Intellectual Negative	New
Lazy	Critical Trait	Intellectual Negative	New
Yellow	Associated Athematic	N/A	New
Marigold	Associated Athematic	N/A	New
Green	Associated Athematic	N/A	New
Closet	Associated Athematic	N/A	New
Furniture	Associated Athematic	N/A	New
Table	Associated Athematic	N/A	New
Pudding	Associated Athematic	N/A	New
Bracelet	Non-Associated Athematic	N/A	New
Boat	Non-Associated Athematic	N/A	New
Calendar	Non-Associated Athematic	N/A	New
Arrogant	Presented Central Trait	Social Negative	New
Understanding	Presented Central Trait	Social Positive	New
Cultured	Presented Central Trait	Intellectual Positive	New
Irresponsible	Presented Central Trait	Intellectual Negative	New
Sensitive	Presented Trait	Social Positive	Old
Kind	Presented Trait	Social Positive	Old
Sociable	Presented Trait	Social Positive	Old
Helpful	Presented Trait	Social Positive	Old
Outgoing	Presented Trait	Social Positive	Old
Intolerant	Presented Trait	Social Negative	Old
Mischievous	Presented Trait	Social Negative	Old
Cold	Presented Trait	Social Negative	Old
Manipulative	Presented Trait	Social Negative	Old
Calculating	Presented Trait	Social Negative	Old
Smart	Presented Trait	Intellectual Positive	Old
Efficient	Presented Trait	Intellectual Positive	Old
Creative	Presented Trait	Intellectual Positive	Old
Rational	Presented Trait	Intellectual Positive	Old
Fighter	Presented Trait	Intellectual Positive	Old
Unable	Presented Trait	Intellectual Negative	Old
Inattentive	Presented Trait	Intellectual Negative	Old
Dependent	Presented Trait	Intellectual Negative	Old
Dumb	Presented Trait	Intellectual Negative	Old
Conservative	Presented Trait	Intellectual Negative	Old
Clock	Presented Athematic	N/A	Old
Count	Presented Athematic	N/A	Old
Telephone	Presented Athematic	N/A	Old

Note: For the presented central traits only one was old, depending on the studied list; and the presented traits included were only the ones from the cluster of the studied list.

APPENDIX 2

Standard Recognition Test Instructions – Self-Paced

"You will be presented with a number of words. Some of these words were included in the audio recording you previously listened to, others are new. Please read each word as it appears in the screen and indicate whether that was included in the audio recording or not. If you recognize the word press the green key (indicating that you consider that the word was included in the audio recording you listened to), but if you think the word is new, press the red key (indicating that you consider that the word was not included in the audio recording). Please press the green key only if you are reasonably certain that the word was included in the audio recording."

Standard Recognition Test Instructions – Time Pressure

"You will be presented with a number of words. Some of these words were included in the audio recording you previously listened to, others are new. Please read each word as it appears in the screen and indicate whether that was included in the audio recording or not. Each word will be presented for one second (1 sec) and you will only have that time to indicate your answer. If you recognize the word press the green key (indicating that you consider that the word was included in the audio recording you listened to), but if you think the word is new, press the red key (indicating that you consider that the word was not included in the audio recording). Please press the green key only if you are reasonably certain that the word was included in the audio recording."
